# Safety and Efficacy of Percutaneous Liver Microwave Ablation Using a Fully Water-Cooled Choke Ring Antenna: First Multicenter Clinical Report

**DOI:** 10.1007/s00270-023-03481-3

**Published:** 2023-07-10

**Authors:** Maxime Blain, Govindarajan Narayanan, Alexis Ricoeur, Adrian Kobe, Ashwin M. Mahendra, Blake Jacks, Quentin Letty, Baptiste Bonnet, Lambros Tselikas, Frederic Deschamps, Thierry de Baère

**Affiliations:** 1 Department of Interventional Radiology, Gustave Roussy, 114 Rue Edouard Vaillant, 94805 Villejuif, France; 2 Herbert Wertheim College of Medicine, Florida International University, Miami, FL USA; 3 Miami Cancer Institute, Baptist Health South Florida, Miami, FL USA; 4 Miami Cardiac and Vascular, Baptist Health South Florida, Miami, FL USA; 5 Interventional Radiology Unit, Diagnostic Department, Geneva University Hospitals, Geneva, Switzerland; 6 Department of Interventional Radiology, Centre Léon Berard, Lyon, France; 7 Charles E. Schmidt College of Medicine, Florida Atlantic University, Miami, FL USA; 8 CARTI Cancer Center, Little Rock, AR USA

**Keywords:** Interventional oncology, Liver neoplasms, Microwave ablation

## Abstract

**Introduction:**

The safety and efficacy of a microwave ablation (MWA) system for the liver with novel technologies in field control, antenna cooling through the inner part of the choke ring, and dual temperature monitoring were evaluated in this multicenter retrospective study.

**Material and Methods:**

Ablation characteristics and efficacy were assessed on follow-up imaging (computed tomography or magnetic resonance imaging). Safety was evaluated according to CTCAE classification.

**Results:**

Eighty-seven liver tumors (65 metastases and 22 hepatocellular carcinomas) measuring 17.8 ± 7.9 mm were treated in 68 patients. Ablation zones measured 35.6 ± 11 mm in longest diameter. The coefficients of variation of the longest and shortest ablation diameters were 30.1% and 26.4%, respectively. The mean sphericity index of the ablation zone was 0.78 ± 0.14. Seventy-one ablations (82%) had a sphericity index above 0.66. At 1 month, all tumors demonstrated complete ablation with margins of 0–5 mm, 5–10 mm, and greater than 10 mm achieved in 22%, 46%, and 31% of tumors, respectively. After a median follow-up of 10 months, local tumor control was achieved in 84.7% of treated tumors after a single ablation and in 86% after one patient received a second ablation. One grade 3 complication (stress ulcer) occurred, but was unrelated to the procedure. Ablation zone size and geometry in this clinical study were in accordance with previously reported in vivo preclinical findings.

**Conclusion:**

Promising results were reported for this MWA device. The high spherical index, reproducibility, and predictability of the resulting treatment zones translated to a high percentage of adequate safety margins, providing good local control rate.

## Introduction

Image-guided thermal ablation (IGTA) provides local control rates close to 90% for liver malignancies [1–3] and is part of the treatment algorithm of primary [4] and secondary malignancies [5]. IGTA technology has evolved from radiofrequency ablation (RFA) to microwave ablation (MWA) and cryoablation [6]].

MWA generators and applicators vary in their applied frequencies (915–2450 MHz), antenna caliber (11–18 G), antenna technology (cooled, choke, sleeves, emission point), generators’ maximum power output (60–200 W), and power loss from generator to antenna emission point [7]. Ablation characteristics and outcomes vary from one MWA system to another [8], warranting the critical appraisal of safety and efficacy for each system.

Therefore, it is important for operators to know the evidence for recent MWA technologies, which aim to generate predictable, reproducible, and spherical ablation zones.

This retrospective review discusses the early clinical assessment of the DOPHI M150E MWA device across three centers. Ablation characteristics of this device are compared with those previously reported by a preclinical study to help readers better define ablation parameters in clinical practice.

## Material and Methods

This multicenter retrospective study included all consecutive patients treated for liver tumors using the DOPHI M150E MWA system (HDTECH, Lorient, France) from June 2020 to July 2021 in 2 hospitals in France and one in the USA. Institutional review board approval was obtained at each participating center.

Liver tumors targeted for ablation were assessed for size and sphericity index (SI), defined as the ratio of shortest diameter (SD) to longest perpendicular diameter (LD) on computed tomography (CT) or magnetic resonance imaging (MRI) obtained within 30 days of the MWA.

MWA was performed under general anesthesia using ultrasound, CT, or robotic navigation (Epione, Quantum surgical, Montpellier, France). Complications were evaluated and reported according to the Common Terminology Criteria for Adverse Events (CTCAE).

**Fig. 1:**
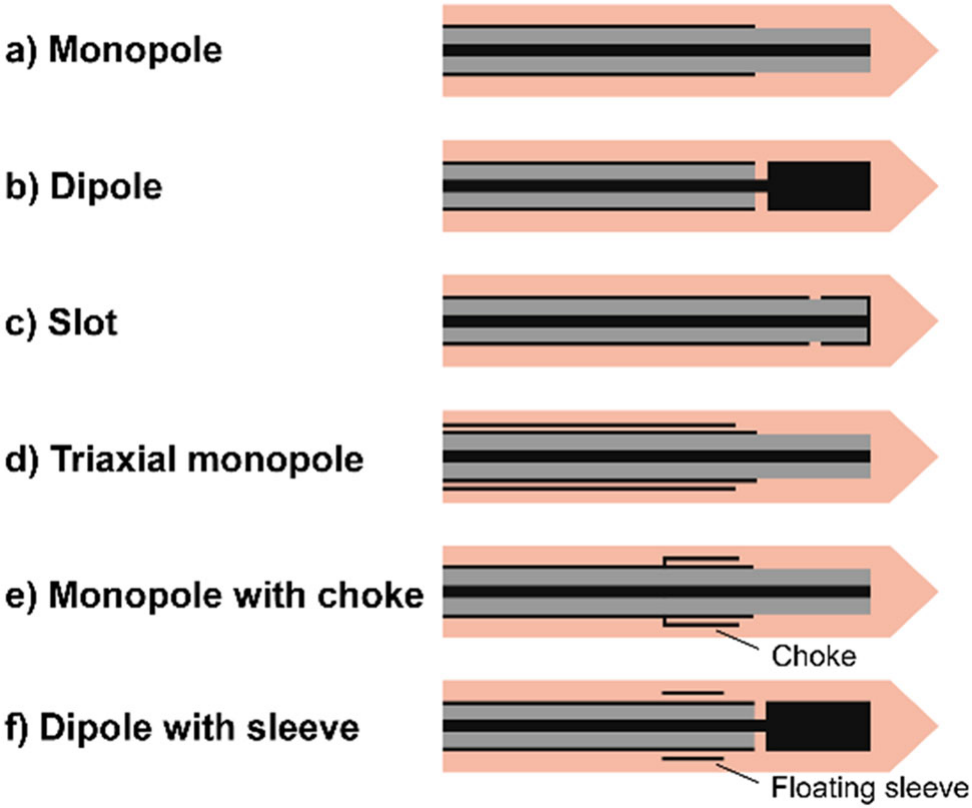
Examples of antenna designs: monopole, dipole, slot, triaxial, choke, and floating sleeve antenna

The Surgnova, Dophi^®^ MWA system uses a unique dipole antenna with floating sleeves that offers advantages over conventional monopolar, dipolar, slot, and triaxial antennas (Fig. 1). These advantages include safeguards against backward heating that causes teardrop or comet-tail-shaped ablation patterns along the feedline of conventional MWA systems. [9–11]. The choke or floating sleeve on the outer conductor of the antenna (Fig. 1) effectively mitigates backward currents along the antenna [11–13]. The floating and choke sleeve antennas serve as backward current suppressors, and the floating sleeve antenna also acts as a radiation part, resulting in higher efficiency. The dipole antenna with floating sleeves offers exceptional localization, high specific absorption rates, and ability to achieve low reflection coefficients or return loss. These features improve the antenna's performance during microwave ablation therapy [14, 15]. Anti-phase^™^ technology built into the antenna ensures that the backward microwave radiation through the outside and inside of the sleeve have opposite phase positions that counteract each other when meeting at the end of the ablation field. This optimizes the distribution of the electromagnetic field to achieve a more spherical ablation area. The Aqua-through™ technology circulates cooling fluid along the entire shaft of the antenna, providing more efficient cooling performance and reducing the antenna temperature during the procedure.

The aim of ablation in this study was to achieve a minimum ablation margin of 5 mm. The ablation protocol was tailored to each tumor according to previously reported in vivo preclinical data [16]. The in vivo preclinical data reported ablation zones of 22 ± 5 × 29 ± 5 mm, 30 ± 6 × 34 ± 8 mm, and 32 ± 4 × 45 ± 6 mm for 50W/5 min, 50W/8 min, and 100W/10 min, respectively [16]. Power emitted and duration of ablation were recorded.

CT or MRI were obtained within 40 days post-ablation to evaluate the size of the ablation zone as defined by LD and SD, sphericity, and ablation margins [17].

CT or MRI obtained at least 60 days post-ablation were used to categorize tumor response by complete ablation, local tumor progression, or distant tumor progression according to standardized terminology and reporting criteria [18].

The coefficient of variation (CV) was calculated as the ratio of the standard deviation to the mean value among different ablations delivered with the same parameters and was provided for both LD and SD. CV was reported only for subgroups that contained at least 8 patients treated with the same ablation parameters (time and power).

Statistical analysis was performed using RStudio 2022.02.3.

## Results

Sixty-eight patients with one (*n* = 54), two (*n* = 9), or three liver tumors (*n* = 5) were treated for a total of 87 liver tumors. Patient and treatment characteristics are described in Table 1.

**Table 1:** Patients population and treatment characteristics. Normal variables are given in mean and SD and non-normal variables are given in median and IQR

Variable	Mean/median	Standard deviation/interquantile range
Age (years)	67	13.5
Nodule longest diameter (mm)	17	9
Nodule short diameter (mm)	13	8
Ablation power (Watts)	75	30
Ablation time (min)	8	4.5
Ablation longest diameter (mm)	34	16
Ablation short diameter (mm)	28	7
Ablation sphericity index	0.78	0.14

	Number
Gender	36 females/32 males
Center	15 Lyon/20 Miami/33 Gustave Roussy
Primary type	18 HCC/18 colorectal/7 breast/5 neuroendocrine/2 corticoadrenaloma/2 pancreas/16 other
Guidance	28 CT/15 CT + US/13 robot/12 US only
Global progression (distant + local)	32 no/36 yes
Local recurrence	58 no/10 yes
Last status	41 alive/6 dead/21 lost

Median follow-up was 10 months (IQR 11), and median progression-free survival was 176.5 days (IQR 269).

**Fig. 2:**
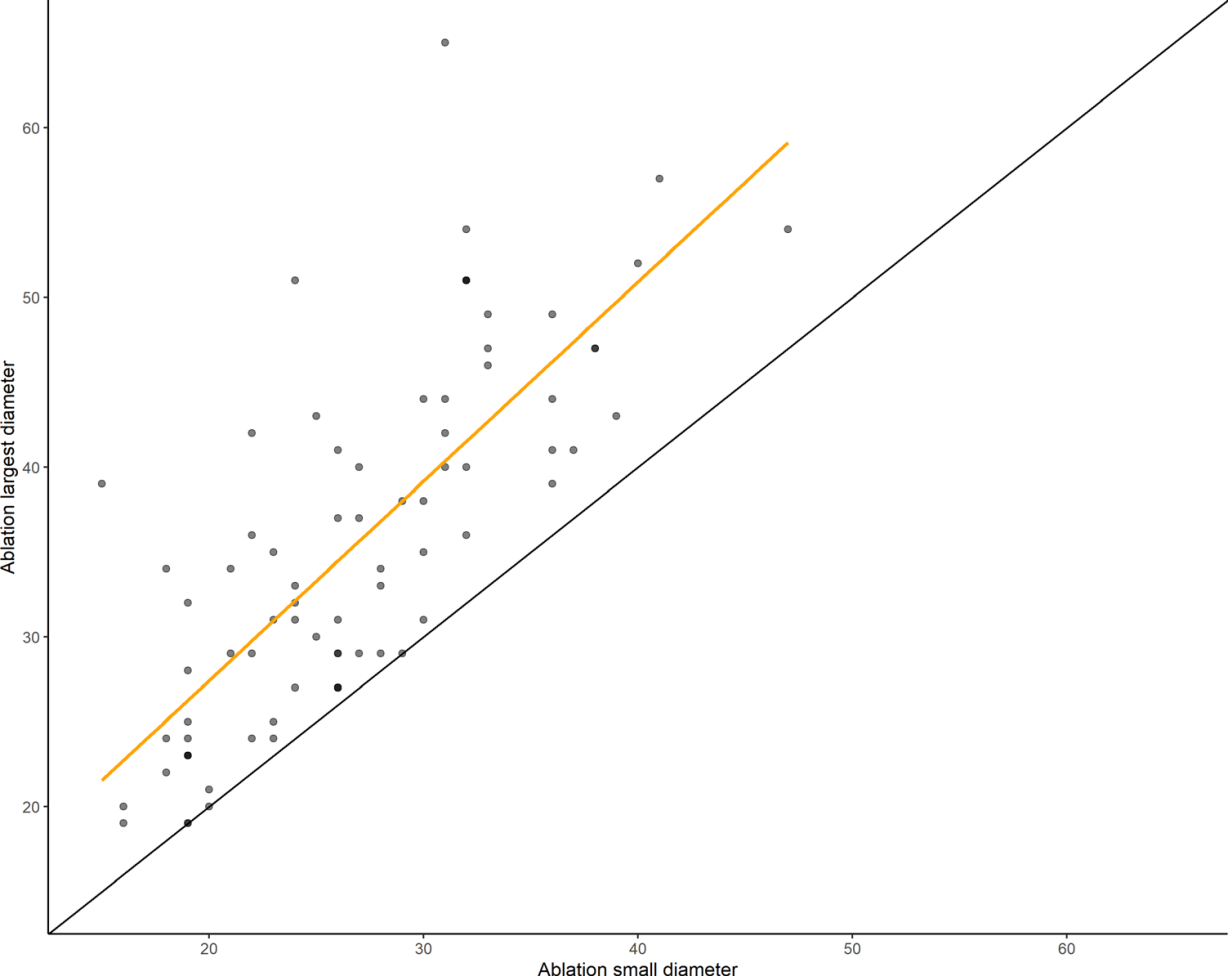
Ablation longest diameter versus ablation smallest diameter. The black continuous line plots perfect sphericity (SI = 1). The linear regression in yellow demonstrates some loss of sphericity for the largest ablation volume

**Fig. 3:**
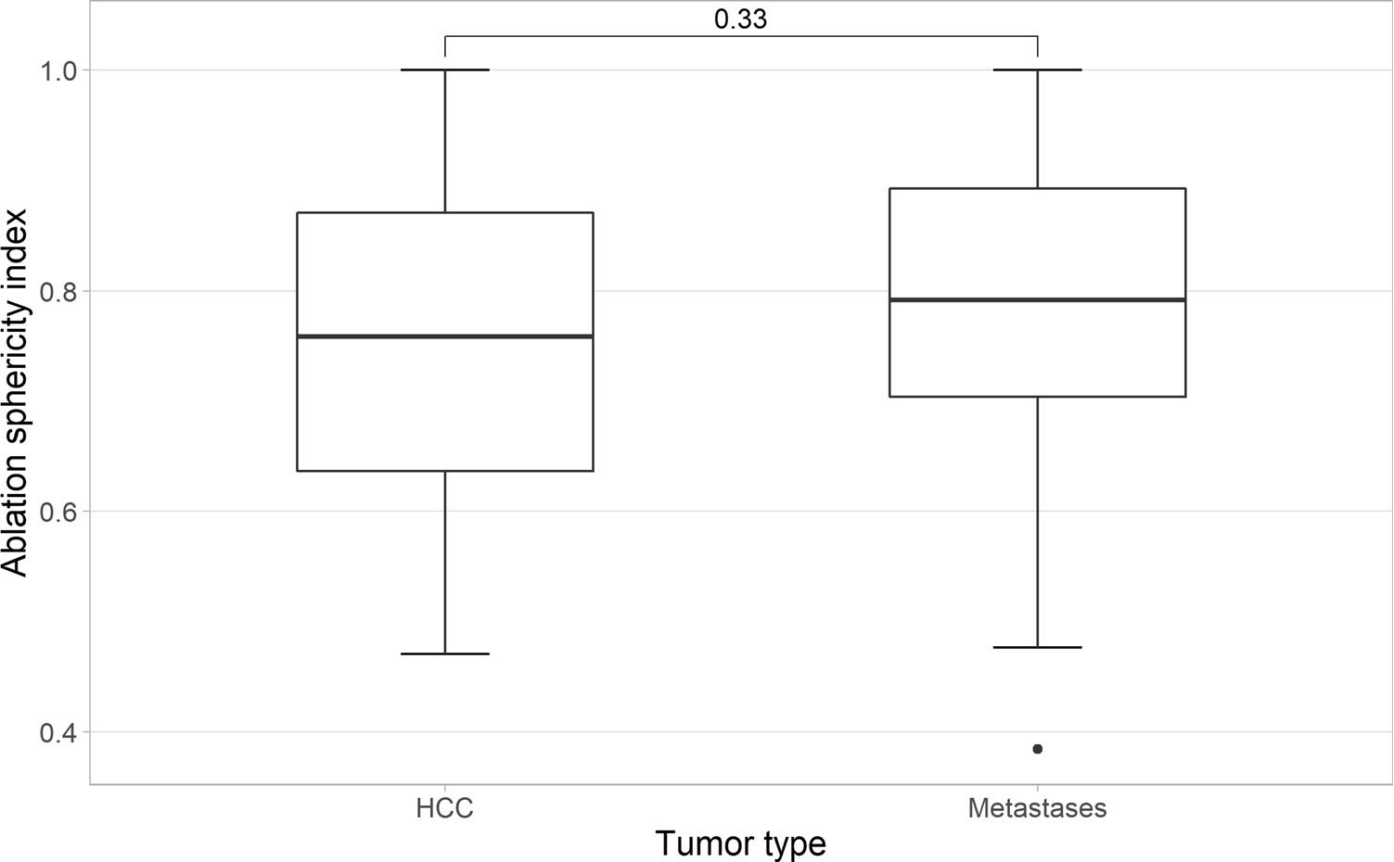
Whisker plot of ablation sphericity depending on tumor type

The median sizes of metastases and hepatocellular carcinoma (HCC) were 21 mm and 15 mm, respectively. Ablation zones measured 35.6 ± 11 in LD and 26.9 ± 7.1 mm in SD in the axial plane. The LD and SD coefficients of variation were 30.1% and 26.4%, respectively. Ablation zones were significantly smaller for metastases than for HCC (33.2 vs 42.6 mm, *p* < 0.05). SI of the ablation zones was 0.78 ± 0.14, with 82% of ablation zones demonstrating a SI above 0.66. SI was not correlated with ablation parameters, including power (*R* = − 0.12, *p* = 0.3) and duration (*R* = − 0.01, *p* = 0.96), even when there was a tendency for less sphericity for a larger ablation volume (Fig. 2). SI was not different between metastases and HCC (0.76 vs 0.73, *p* = 0.33) (Fig. 3). CV ranged from 0.22 to 0.33 for LD and 0.17 to 0.26 for SD for the most populated subgroups (Table 2). Variations between these results and those of the previously published in vivo preclinical study [16] using the same MWA system were small and decreased with the increase in the applied energy (Table 3).

**Table 2:** Coefficient of variation for longest and short diameters calculated for the most populated subgroups

Subgroup	Longest diameter	Short diameter
50W 5 min	0.32	0.25
50W 8 min	0.22	0.26
100W 8 min	0.22	0.20
75W 10 min	0.33	0.17
100W 10 min	0.28	0.26

**Table 3:** Differences in ablation sizes at given power and time between the herein reported clinical study and in vivo preclinical study previously published [9]

	Longest diameter difference (mm)		Shortest diameter difference (mm)	
	Median	Mean	Median	Mean
50W 5 min	− 7.5	− 4.75	− 3.5	− 3.125
50W 8 min	− 6	− 3.286	− 9	− 8.143
100W 8 min	3	1.455	2	− 0.18
75W 10 min	− 5.5	− 0.917	0	0.5
100W 10 min	3	− 0.3	0.5	1

At 1-month follow-up imaging, all tumors demonstrated complete ablation. One grade 3 complication was reported—a bleed from a stress ulcer considered unrelated to the MWA. One minor hepatic subcapsular hematoma not requiring intervention was noted during a procedure.

Seventy-two treated tumors in 50 patients met the minimum follow-up of 2 months (median: 378 days; IQR 264) for assessment of tumor response, demonstrating a primary local tumor control rate of 84.7%, and a secondary local tumor control rate of 86% after one patient received a second ablation. The rate of distant progression was 72%.

Among the 11 patients who experienced local tumor progression, two had ablation margins greater than 10 mm, four had margins between 5 and 10 mm, and 5 had margins under 5 mm. Among the 72 treated tumors with minimum follow-up of 2 months, 20 tumors (28%) had ablation margins greater than 10 mm, 30 tumors (42%) had margins between 5 and 10 mm, and 18 tumors (25%) had margins between 0 and 5 mm.

## Discussion

This multicenter clinical study describing the use of the DOPHI M150E MWA unit for liver ablation margins of over 5 mm was achieved in 78% of treated tumors with a local tumor control rate of 86% after a median follow-up over 1 year for tumors 17.8 ± 7.9 mm in size. These outcomes are promising and consistent with the best published results of IGTA [19, 20]. The low complication rate confirms the safety of this relatively new MWA system.

The size and shape of ablation obtained in the clinical study matched with the data reported in preclinical work using the same MWA system [16], with a difference in ablation zone dimensions of under 1.5 mm when working at power above 50 W (Table 3). The ablation zone CV in this clinical study was within 0.1 of the values in the preclinical study at 50W/5 min, 50W/8 min, and 100W/8 min [16]. The observed increase in CV with longer treatment duration is most likely a consequence of heterogeneity in tumor type and underlying liver parenchyma in clinical practice versus the more homogeneous livers used in the preclinical study. It is known that when ablation time increases, there is more passive rather than active heating [21, 22]. Thus, greater variation in heating may develop due to thermal conduction and convection of heterogeneous tissues. These findings favor the use of short, high-power ablations versus long, low-power ablations for the sake of reproducibility. SI in the present work was similar to those in the preclinical study with a median of 0.61 versus 0.58, respectively. This is likely due to features of the antenna design described earlier that play a crucial role in optimizing outcomes.

Previously published ablation charts obtained in animals offer a valid basis for the selection of ablation parameters in liver applications for clinical practice. The ablation zones of liver tumors for the same energy deposit were larger for HCC than metastases. This may be explained by liver cirrhosis modifying the heat conduction in the surrounding liver tissue. Therefore, lowering the energy deposited by reducing the ablation time or power may be required in the treatment of cirrhotic livers when compared to the preclinical data. Also, the linear regression of the curve tends to diverge from perfect sphericity when comparing the SD and LD for higher energy and larger ablation zones. This relationship did not achieve statistical significance, but may imply that, for a given amount of energy delivered, lower power and more prolonged treatment time achieve more spherical ablation zones.

Our study has some limitations, including its retrospective nature, relatively low number of participants (considering the heterogeneity in tumor type and ablation protocol), and the lack of a control group treated with a more conventional and widely used MWA device.

## Conclusion

Overall, the results of this first multicenter clinical study with the DOPHI system achieved acceptable control rates for both HCC and liver metastases. The study also establishes the safety and efficacy of the DOPHI system, validates the results of the previously published preclinical data and highlights the importance of extensive in vivo preclinical experimentation before use of any specific microwave system in clinical practice.
